# The RNA-binding protein MARF1 promotes cortical neurogenesis through its RNase activity domain

**DOI:** 10.1038/s41598-017-01317-y

**Published:** 2017-04-25

**Authors:** Yoshitaka Kanemitsu, Masashi Fujitani, Yuki Fujita, Suxiang Zhang, You-Qiang Su, Yukio Kawahara, Toshihide Yamashita

**Affiliations:** 1 Department of Molecular Neuroscience, Graduate School of Frontier Biosciences, Osaka University, 1-3 Yamadaoka, Suita, Osaka 565-0871 Japan; 2 Interdisciplinary Program for Biomedical Sciences, Osaka University, 2-2 Yamadaoka, Suita, Osaka 565-0871 Japan; 3 Department of Molecular Neuroscience, Graduate School of Medicine, Osaka University, 2-2 Yamadaoka, Suita, Osaka 565-0871 Japan; 4 Molecular Research Center for Children’s Mental Development, United Graduate School of Child Development, Osaka University, 2-2 Yamadaoka, Suita, Osaka 565-0872 Japan; 5 Department of Anatomy and Neuroscience, Hyogo College of Medicine, 1-1, Mukogawa-cho, Nishinomiya, Hyogo 663-8501 Japan; 6 State Key Laboratory of Reproductive Medicine, Nanjing Medical University, 140 Hanzhong Road, Nanjing, 210029 Jiangsu Province China; 7 Department of RNA Biology and Neuroscience, Graduate School of Medicine, Osaka University, 2-2 Yamadaoka, Suita, Osaka 565-0871 Japan

## Abstract

Cortical neurogenesis is a fundamental process of brain development that is spatiotemporally regulated by both intrinsic and extrinsic cues. Although recent evidence has highlighted the significance of transcription factors in cortical neurogenesis, little is known regarding the role of RNA-binding proteins (RBPs) in the post-transcriptional regulation of cortical neurogenesis. Here, we report that meiosis arrest female 1 (MARF1) is an RBP that is expressed during neuronal differentiation. Cortical neurons expressed the somatic form of MARF1 (sMARF1) but not the oocyte form (oMARF1). sMARF1 was enriched in embryonic brains, and its expression level decreased as brain development progressed. Overexpression of sMARF1 in E12.5 neuronal progenitor cells promoted neuronal differentiation, whereas sMARF1 knockdown decreased neuronal progenitor differentiation *in vitro*. We also examined the function of sMARF1 *in vivo* using an *in utero* electroporation technique. Overexpression of sMARF1 increased neuronal differentiation, whereas knockdown of sMARF1 inhibited differentiation *in vivo*. Moreover, using an RNase domain deletion mutant of sMARF1, we showed that the RNase domain is required for the effects of sMARF1 on cortical neurogenesis *in vitro*. Our results further elucidate the mechanisms of post-transcriptional regulation of cortical neurogenesis by RBPs.

## Introduction

Cortical neurogenesis is a fundamental process of brain development that is precisely regulated throughout development by a variety of intrinsic and extrinsic cues in cortical progenitor cells^1^. Although a number of transcription factors and signaling molecules are involved in cortical neurogenesis, the roles of RNA-binding proteins (RBPs) in cortical development have only begun to be elucidated^2^. RBPs bind to target RNAs and regulate post-transcriptional events, such as alternative splicing, translation and mRNA decay^3^. A comprehensive analysis of the expression of RBPs in the human brain suggested that approximately 100 RBPs are highly expressed during the early developmental stage^4^; however, only a small number of RBPs have been investigated for a possible functional role during corticogenesis^2^. Well-documented roles of RBPs in progenitor cells include the regulation of alternative splicing^5–7^, translation^8–13^, and RNA localization^14, 15^. Interestingly, no studies have reported a role for RBPs in mRNA decay during cortical development^2^.

Meiosis arrest female 1 (MARF1) is an RBP that contains a Nedd4-BP1 domain and a YacP nuclease (NYN)/PilT N-terminus (PIN)-like domain, which displays RNase activity^16, 17^. MARF1 has two isoforms, an oocyte form (oMARF1) and a somatic form (sMARF1). The two splice variants have the same sequence except exon 3. sMARF1 has a longer exon 3 (901 bp) than oMARF1 (364 bp)^16^. oMARF1 is highly expressed in mouse oocytes and regulates the oogenic process by silencing the expression of *Ppp2cb* ^16^. We previously reported that *Marf1* mRNA is expressed in a limited fashion in the developing mouse brain^18^. This finding led us to hypothesize that MARF1 has an essential role in cortical development.

Here, we showed that sMARF1 is expressed in the developing cortex and that its expression increases with neuronal differentiation. Our gain-of-function and loss-of-function experiments revealed that sMARF1 promotes neuronal differentiation both *in vivo* and *in vitro*.

## Results

### Somatic form of *Marf1* is expressed in the developing cortex

**Figure 1:**
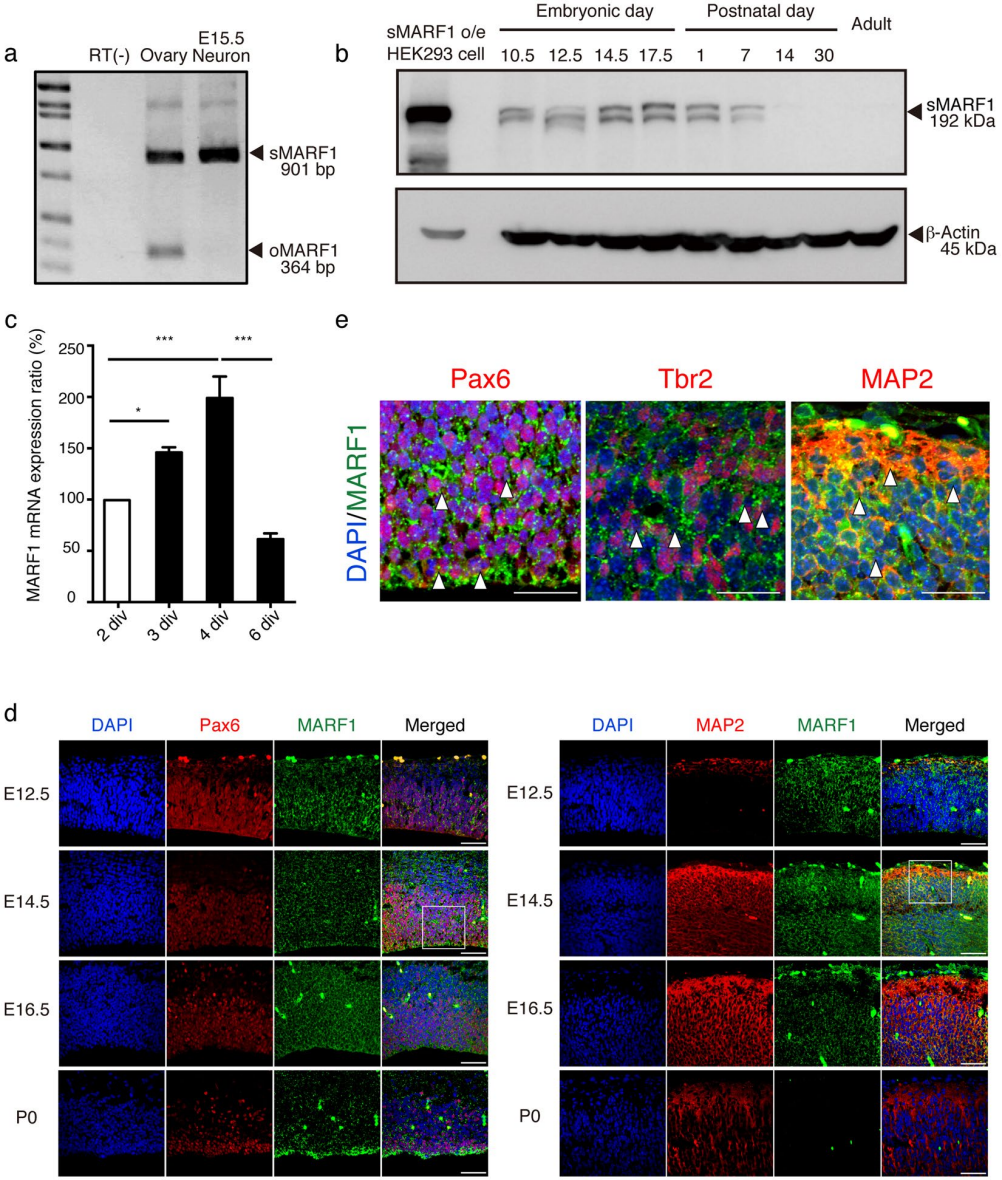
The somatic form of *Marf1* (*sMarf1*) is expressed in the developing cortex. (**a**) RT-PCR analysis for exon 3 of *Marf1* mRNA in mouse ovary tissue and E15.5 cultured cortical neurons. The somatic form of *Marf1* (sMARF1) contains a longer exon 3 (901 base pairs) than the ovarian form (oMARF1: 364 base pairs). The full-length gel is presented in Supplementary Fig. 8a. (**b**) Western blotting for sMARF1 in mouse cortical lysates at the embryonic, postnatal, and adult ages. β-Actin was used as a loading control. Lysate of HEK293 cells transfected with a *sMarf1* expression vector was used as a positive control. The same full-length image is presented in Supplementary Fig. 8b, because membrane was cut before incubating with antibodies. (**c**) Relative expression levels of *Marf1* mRNA from E12.5 cortical progenitors cultured for 2, 3, 4 and 6 days (2, 3, 4 and 6 days *in vitro* (div)). *p < 0.05 ***p < 0.001 (n = 4, one-way ANOVA, Tukey-Kramer test). (**d**) Developmental expression change of sMARF1. Immunostaining for MAP2 (red) or Pax6 (red), DAPI (blue), and MARF1 (green) in coronal sections of the E12.5, 14.5, 16.5 and postnatal day 0 mouse cortices. Scale bar, 50 μm. (**e**) Magnified images of E14.5 mouse cortex immunostained for Pax6, Tbr2 or MAP2. White arrowheads indicate MARF1 and Pax6^+^ radial glia (left-panel), MARF1 and Tbr2 co-expressing differentiated basal progenitors (middle-panel), or MARF1 and MAP2^+^ post-mitotic neurons (right-panel). Scale bar, 25 μm.

We previously found that *Marf1* mRNA is expressed in the embryonic and postnatal brain^18^. In addition, *Marf1* occurs as two isoforms: *sMarf1* and *oMarf1* ^16^. To determine which isoform of *Marf1* is expressed in cortical neurons, we performed RT-PCR analysis of embryonic primary neurons using ovary tissue as a positive control. E15.5 cortical neurons expressed sMARF1 but not oMARF1 (Fig. 1a). To examine the sMARF1 expression profile during various developmental stages, we performed western blot analysis of cortical brain samples obtained from mice at different stages of development (E10.5, E12.5, E14.5, E17.5, P1, P7, P14, P30, and adult) using a MARF1-specific antibody^16^. We found that sMARF1 expression was prominent from E14.5 to E17.5 and gradually decreased in the adult stages (Fig. 1b). To determine whether *sMarf1* expression levels changed depending on the cellular differentiation state, we performed an *in vitro* differentiation assay using primary E12.5 cortical progenitor cells^18^. We first confirmed the differentiation time frame of progenitor cells to neurons by differentiation assays (Supplementary Fig. 1a and b). We observed that *sMarf1* mRNA increased gradually as cortical progenitor cells differentiated to neurons *in vitro*, but its expression finally declined after 6 days (Fig. 1c). To confirm these findings *in vivo*, we performed immunostaining of sMARF1 with MAP2 (neuronal marker), Tbr2 (basal progenitor marker) or Pax6 (radial glial marker) in E12.5, E14.5, E16.5 and P0 mouse cortices. The expression of sMARF1 was detected in E12.5, E14.5, E16.5, and P0 cortices (Fig. 1d and Supplementary Figs 3, 4, 5 and 6). Pax6^+^ radial glia, Tbr2^+^ basal progenitors and MAP2^+^ neurons co-expressed sMARF1 in the E14.5 cortex (Fig. 1e). However, after reaching to the highest expression at approximately E16.5, MAP2^+^ neuronal expression decreased at P0, as observed in the *in vitro* expression analysis. These results suggest that sMARF1 is involved in early to mid-term brain development presumably during progenitor differentiation to neurons.

### sMARF1 promotes differentiation of cortical progenitor cells *in vitro*

**Figure 2:**
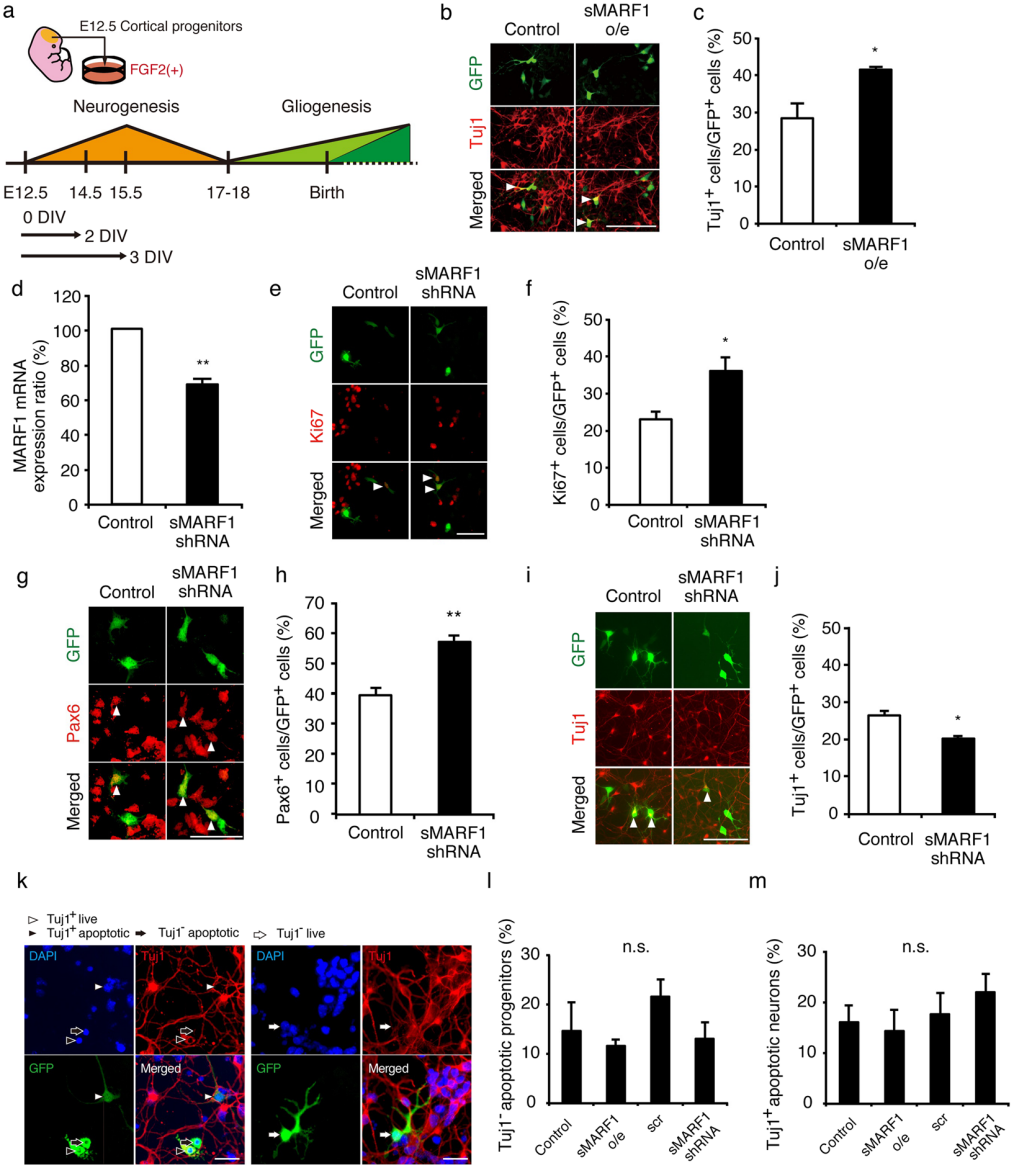
sMARF1 promotes the differentiation of cortical progenitor cells *in vitro*. (**a**) Schematic of the *in vitro* culture system of E12.5 mouse cortical progenitor cells. (**b**) Immunostaining for GFP (green) and Tuj1 (red) in E12.5 cortical progenitor cells 3 days after transfection with pCAGIG (Control) or an *sMarf1* expression vector (sMARF1 o/e). White arrowheads indicate GFP-labeled Tuj1^+^ post-mitotic neurons. Scale bar, 50 μm. (**c**) Quantification of the GFP-labeled Tuj1^+^ cells. *p < 0.05 (n = 3, Student’s *t*-test). (**d**) Expression of *Marf1* mRNA in E15.5 cortical neurons 2 days after nucleofection with scrambled (Control) or *Marf1*-specific shRNA vector (sMARF1 shRNA). **p < 0.01 (n = 3, Student’s *t*-test). (**e**) Immunostaining for GFP (green) and Ki67 (red) in E12.5 cortical progenitor cells 2 days after transfection with scrambled (Control) or *Marf1*-specific shRNA vector (sMARF1 shRNA). White arrowheads indicate GFP-labeled Ki67^+^ progenitor cells. Scale bar, 50 μm. (**f**) Quantification of the GFP-labeled Ki67^+^ cells. *p < 0.05 (n = 3, Student’s *t*-test). (**g**) Immunostaining for GFP (green) and Pax6 (red) in E12.5 cortical progenitor cells 2 days after transfection with scrambled (Control) or *Marf1*-specific shRNA vector (sMARF1 shRNA). White arrowheads indicate GFP-labeled Pax6^+^ radial glial cells. Scale bar, 50 μm. (**h**) Quantification of the GFP-labeled Pax6^+^ cells. **p < 0.01 (n = 3, Student’s *t*-test). (**i**) Immunostaining for GFP (green) and Tuj1 (red) in E12.5 cortical progenitor cells 3 days after transfection with scrambled (Control) or *Marf1*-specific shRNA vector (sMARF1 shRNA). White arrowheads indicate GFP-labeled Tuj1^+^ post-mitotic neurons. Scale bar, 50 μm. (**j**) Quantification of the GFP-labeled Tuj1^+^ cells. *p < 0.05 (n = 3, Student’s *t*-test). (**k**) Immunostaining for DAPI (blue), GFP (green) and Tuj1 (red). White arrowheads indicate Tuj1^+^ living neurons. Black arrowheads indicate Tuj1^+^ apoptotic neurons. Black arrows indicate Tuj1^−^ apoptotic progenitors. (Left panels) White arrows indicate Tuj1^−^ living progenitors. (Right panels) Scale bar, 50 μm. (**l,m**) Quantification of the apoptotic GFP-labeled Tuj1^−^ progenitors (**l**) or Tuj1^+^ neurons (**m**) transfected with pCAGIG (Control), *sMarf1* expression vector (sMARF1 o/e), scrambled (scr) or *Marf1*-specific shRNA vector (sMARF1 shRNA) (n = 3, one-way ANOVA, Tukey-Kramer test).

To determine whether sMARF1 controls the proliferation and differentiation of cortical progenitors, we performed *in vitro* gain- and loss-of-function experiments using E12.5 cortical progenitor cells, as previously described (Fig. 2a)^19^. For the gain-of-function experiments, we transfected cells with an *sMarf1* overexpression construct, and for the loss-of-function experiments, we transfected cells with small-hairpin RNA (shRNA) vectors against *sMarf1*. We first confirmed the knockdown efficiency of *sMarf1* in cortical neurons (Fig. 2d). Overexpression of *sMarf1* in E12.5 cortical progenitor cells increased the percentage of Tuj1^+^ neurons compared with the control cells (Fig. 2b and c). Next, knockdown of *sMarf1* increased the percentage of Ki67^+^ progenitor cells compared with the control cells (Fig. 2e and f). In addition, knockdown of *sMARF1* increased the percentage of Pax6^+^ radial glia but decreased Tuj1^+^ neurons compared with the controls (Fig. 2g,h,i and j). To exclude the possibilities of shRNA vector toxicity and the effect of sMARF1 on the cell survival, we assessed the effect of sMARF1 on the cell death of progenitors and neurons by cell death assays. Transfecting scrambled shRNA vector into progenitors (scr), knockdown of *sMarf1* (sMARF1 shRNA) or overexpression of sMARF1 (sMARF1o/e) did not significantly affect progenitor or neuronal survival (Fig. 2k,l and m). These results indicate that sMARF1 promotes the differentiation of cortical progenitor cells but does not affect cell survival.

### sMARF1 regulates the neuronal differentiation of radial glia in the embryonic cortex *in vivo*

**Figure 3:**
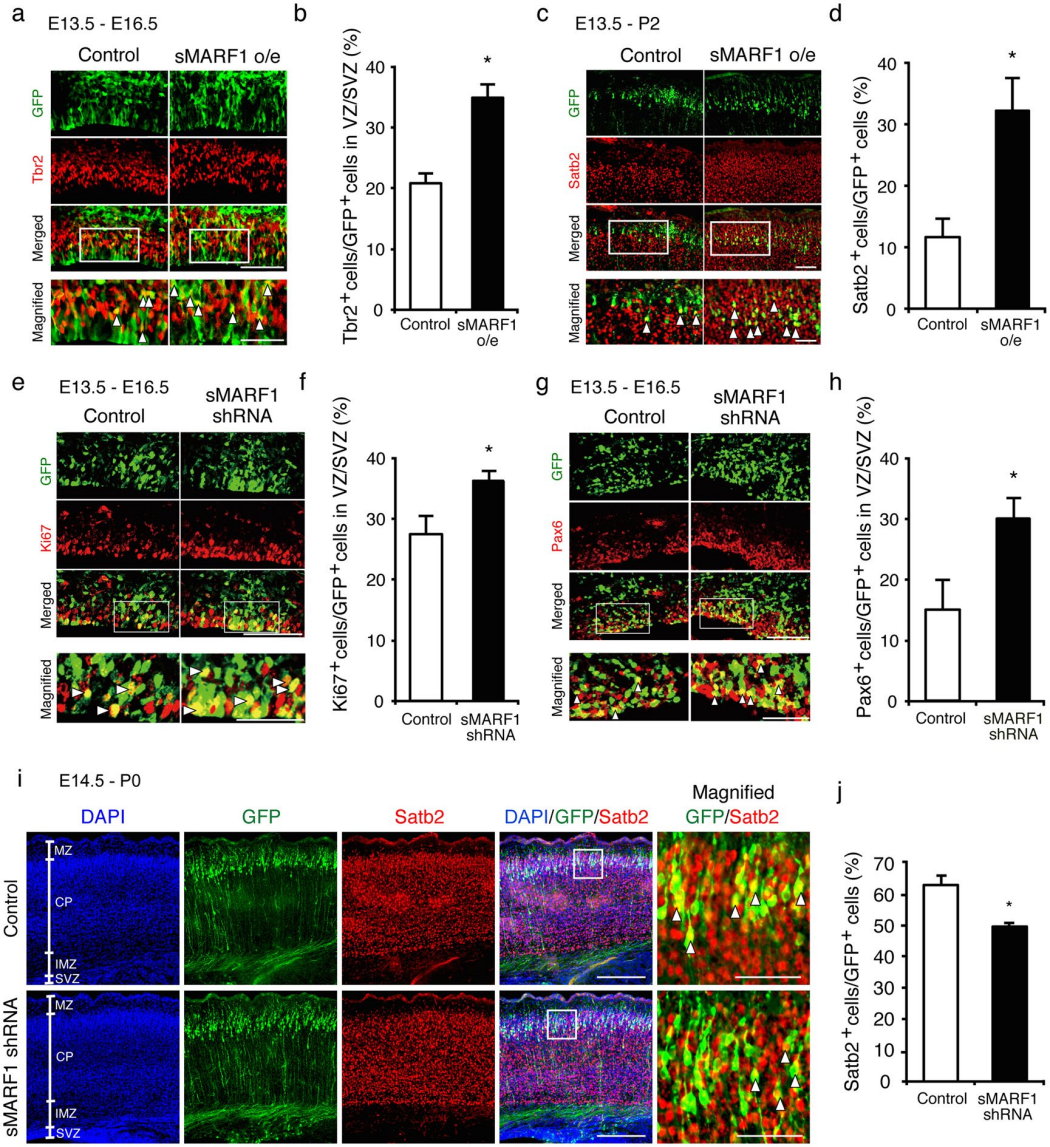
sMARF1 regulates cortical progenitor differentiation *in vivo*. (**a**) Immunostaining for GFP (green) and Tbr2 (red) at the SVZ in E16.5 mouse cortices 3 days after electroporation with pCAGIG (Control) or *sMarf1* expression vector (sMARF1 o/e). White arrowheads indicate GFP-labeled Tbr2^+^ basal progenitor cells. Scale bar, 100 μm in the merged image, 50 μm in the magnified image. (**b**) Quantification of GFP-labeled Tbr2^+^ cells in the VZ and SVZ (VZ/SVZ). *p < 0.05 (n = 3, Student’s *t*-test). (**c**) Immunostaining for GFP (green) and Satb2 (red) at the upper-layer of postnatal day 2 mouse cortices 7 days after electroporation with pCAGIG (Control) or *sMarf1* expression vector (sMARF1 o/e). White arrowheads indicate GFP-labeled Satb2^+^ upper-layer neurons. Scale bar, 400 μm in the merged image, 200 μm in the magnified image. (**d**) Quantification of GFP-labeled Satb2^+^ cells in the upper-layer cortex. *p < 0.05 (n = 3, Student’s *t*-test). (**e**) Immunostaining for GFP (green) and Ki67 (red) in the SVZ of E16.5 mouse cortices 3 days after electroporation with scrambled (Control) or *Marf1*-specific shRNA vector (sMARF1 shRNA). White arrowheads indicate GFP-labeled Ki67^+^ cells. Scale bar, 100 μm in the merged image, 50 μm in the magnified image. (**f**) Quantification of GFP-labeled Ki67^+^ cells in the VZ/SVZ. *p < 0.05 (n = 3, Student’s *t*-test). (**g**) Immunostaining for GFP (green) and Pax6 (red) at the SVZ in E16.5 mouse cortices 3 days after electroporation with scrambled (Control) or *Marf1*-specific shRNA vector (sMARF1 shRNA). White arrowheads indicate GFP-labeled Pax6^+^ radial glia. Scale bar, 100 μm in the merged image, 50 μm in the magnified image. (**h**) Quantification of GFP-labeled Pax6^+^ cells in the VZ/SVZ. *p < 0.05 (n = 3, Student’s *t*-test). (**i**) Immunostaining for GFP (green) and Satb2 (red) at the upper-layer of postnatal day 0 mouse cortices 5 days after electroporation with scrambled (Control) or *Marf1*-specific shRNA vector (sMARF1 shRNA). White arrowheads indicate GFP-labeled Satb2^+^ upper-layer neurons. Scale bar, 200 μm in the merged image, 50 μm in the magnified image. MZ: marginal zone, CP: cortical plate, IMZ: intermediate zone, SVZ: subventricular zone (**j**) Quantification of GFP-labeled Satb2^+^ cells in the upper-layer cortex. *p < 0.05 (n = 3, Student’s *t*-test).

To confirm the function of sMARF1 *in vivo*, we conducted *in utero* electroporation experiments. First, we introduced *sMarf1* expression vectors containing an internal ribosome entry site (IRES)-green fluorescent protein (GFP) expression cassette into E13.5 mouse cortices. Then, the cortices were dissected at E16.5, sectioned and stained for sMARF1 to confirm its overexpression (Supplementary Fig. 2a). To analyze differentiation of radial glia to basal progenitors (BPs), we performed immunostaining for the BP marker Tbr2 as well as GFP (Fig. 3a). *sMarf1* overexpression increased the ratio of GFP-labeled Tbr2^+^ cells within the ventricular zone (VZ) and the subventricular zone (SVZ) (VZ/SVZ) (Fig. 3b). Next, we examined the longer-term effect of sMARF1 expression on cortical progenitor cells. To this end, we performed *in utero* electroporation at E13.5 and then immunostained for the upper-layer neuronal marker Satb2 as well as GFP at P2 and 7 days after electroporation (Fig. 3c). *sMarf1* overexpression increased the percentage of GFP and Satb2 double-positive neurons compared with the control mice (Fig. 3d). These results indicate that sMARF1 promotes cortical radial glial differentiation to BPs and upper-layer neurons. For loss-of-function experiments *in vivo*, we conducted *in utero* electroporation of our shRNA vector as well as that of a nuclear GFP expression vector into E13.5 mouse cortices. First, the cortices were immunostained for GFP and MARF1 at E16.5, and we confirmed protein knockdown by shRNA vector in the brains (Supplementary Fig. 2b). Then, we immunostained brain sections for GFP and Ki67 or Pax6 (Fig. 3e and g). *sMarf1* knockdown increased the ratio of GFP-labeled Ki67^+^ progenitor cells and Pax6^+^ radial glia within the VZ/SVZ compared with the control mice (Fig. 3f and h). To analyze the longer-term effect of *sMarf1* knockdown, we electroporated the shRNA vector and nuclear GFP into E14.5 mouse cortices and then immunostained for Satb2 and GFP at P0 (Fig. 3i). *sMarf1* knockdown decreased the ratio of GFP-labeled Satb2^+^ upper-layer neurons compared with the control (Fig. 3j). Moreover, to determine whether the gain- and loss-of-function of sMARF1 alters the neuronal laminar distribution, we analyzed P0 mouse cortices electroporated with the sMARF1 expression vector or shRNA vector at E14.5 by immunostaining for DAPI, GFP and Satb2 (Fig. 3i and Supplementary Fig. 7). However, gain- or loss-of-sMARF1 had no effect on laminar distribution compared with the control. Taken together, these results suggest that sMARF1 promotes the neuronal differentiation of radial glia *in vivo*, consistent with the *in vitro* observations.

### The RNase activity domain of sMARF1 is necessary for neuronal differentiation

Previous reports have indicated that the NYN/PIN-like domain of oMARF1 exhibits RNase activity^16^. The sequence difference between oMARF1 and sMARF1 occurs outside the NYN/PIN-like domain. The amino acid sequences of the NYN/PIN-like domains are same between both isoforms without frameshifts. These observations suggest that protein function may be conserved between these two isoforms.

**Figure 4:**
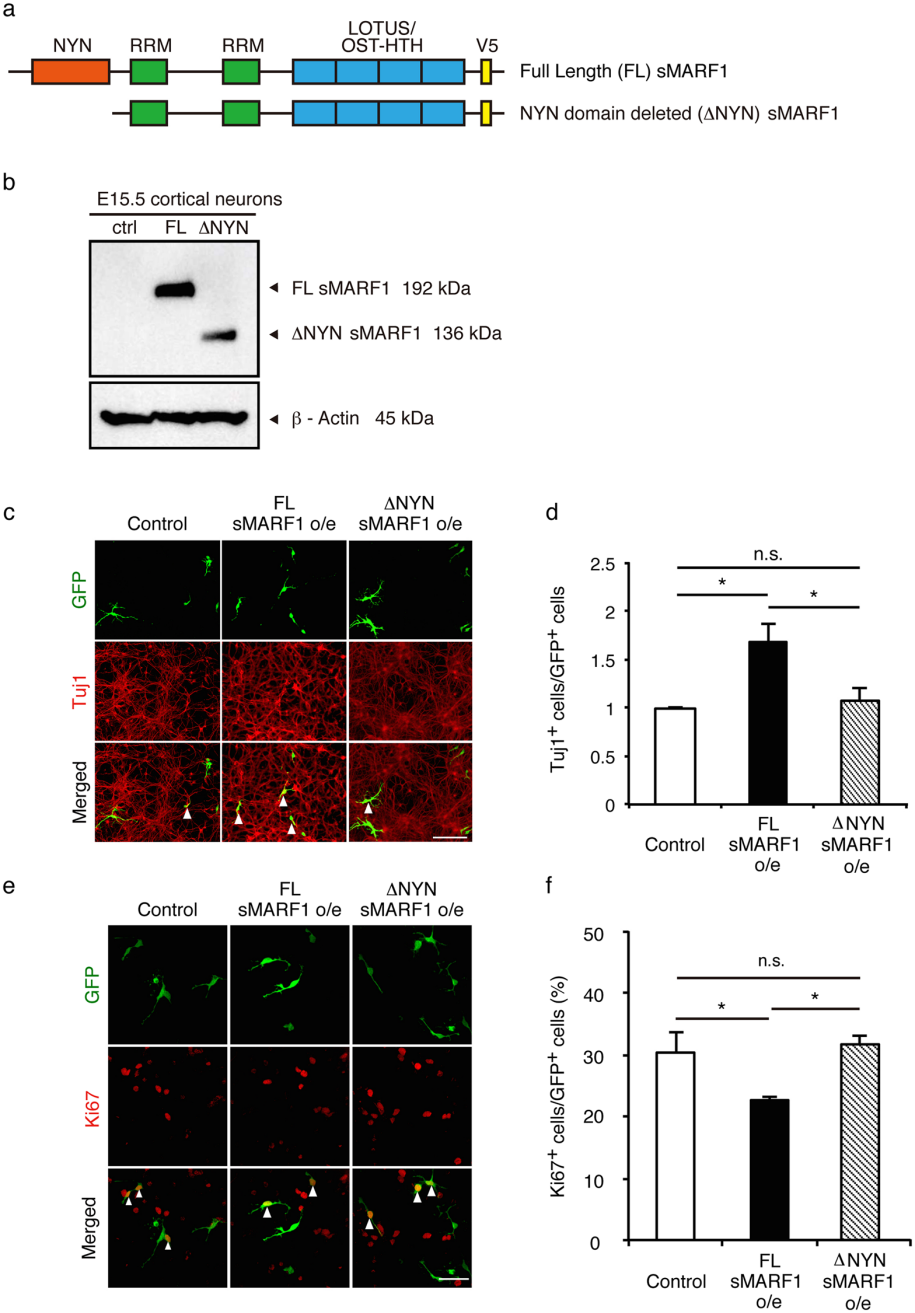
The RNase activity domain of sMARF1 is necessary for neuronal differentiation. (**a**) Domain composition of full-length (FL) sMARF1 and NYN/PIN-like domain deleted (ΔNYN) sMARF1 with carboxy-terminal V5 epitope tags. RRM; RNA recognition motif, OST-HTH; Oskar-TDRD5/TDRD7-helix-turn-helix^39^. (**b**) Western blotting for V5 epitope in E15.5 cortical neurons transfected with pCAGIG (ctrl), FL sMARF1 expression vector (FL), or ΔNYN sMARF1 expression vector (ΔNYN). β-Actin was used as a loading control. The full-length membrane is presented in Supplementary Fig. 8c. (**c**) Immunostaining with GFP (green) and Tuj1 (red) in E12.5 cortical progenitors 3 days after transfection with pCAGIG (Control), FL sMARF1 expression vector (FL sMARF1 o/e), or ΔNYN sMARF1 expression vector (ΔNYN sMARF1 o/e). Scale bar, 100 μm. (**d**) Quantification of GFP-labeled Tuj1^+^ cells. *p < 0.05 (n = 4, one-way ANOVA Tukey-Kramer test). (**e**) Immunostaining with GFP (green) and Ki67 (red) in E12.5 cortical progenitors 2 days after transfection with pCAGIG (Control), FL sMARF1 expression vector (FL sMARF1 o/e), or ΔNYN sMARF1 expression vector (ΔNYN sMARF1 o/e). Scale bar, 100 μm. (**f**) Quantification of GFP-labeled Ki67^+^ cells. *p < 0.05 (n = 3, one-way ANOVA, Tukey-Kramer test).

To determine whether the NYN/PIN-like domain is necessary for neuronal differentiation, we deleted the NYN/PIN-like domain (ΔNYN) in an *sMarf1* expression vector (Fig. 4a and b). For analysis of the effect of this expression vector on cortical progenitor differentiation, we overexpressed full-length (FL) sMARF1 and ΔNYN sMARF1 (Fig. 4c and e) in cortical progenitor cells *in vitro*. Although FL sMARF1 overexpression promoted the differentiation and inhibited the proliferation of progenitor cells, ΔNYN sMARF1 overexpression did not affect either differentiation or proliferation (Fig. 4d and f). Therefore, these results suggest that sMARF1 may induce neuronal differentiation through its NYN/PIN-like domain *in vitro*.

## Discussion

In the present study, we found that cortical neurons expressed the somatic isoform of *Marf1* (*sMarf1*) but not the oocyte form (*oMarf1*). The expression of sMARF1 protein peaked between E14.5 and E17.5, after which it was downregulated in later postnatal stages and adult tissues. sMARF1 levels increased and finally declined to control levels as cells differentiated from cortical progenitors to mature neurons *in vitro*. In the E14.5 brain, not only post-mitotic neurons, but also progenitors, highly expressed sMARF1. Overexpression of sMARF1 promoted the differentiation of cortical progenitor cells to neurons, whereas knockdown of sMARF1 increased the proliferation of progenitor cells without influencing progenitor and neural survival. Transfection of sMARF1 lacking the domain containing RNase activity completely abolished the pro-differentiation activity of sMARF1. These results strongly suggest that sMARF1 acts as a pro-neural protein through its N-terminal RNase activity domain.

The sMARF1 protein expression is prominent from early to mid-term brain development during progenitor differentiation to neurons. Importantly, sMARF1 expression is decreased in the fully differentiated neurons *in vitro* and *in vivo* at later stages. This expression change may be consistent with the hypothesis that sMARF1 is specifically required for neuronal differentiation. As shown in Fig. 2k,l and m, Fig. 3i and Supplementary Fig. 7, cell survival or neuronal migration was not affected by sMARF1 knockdown and overexpression. These results also support the idea that sMARF1 specifically regulates progenitor differentiation.

As shown in Fig. 3i, although the Satb2^+^ neuronal differentiation rate was decreased, laminar distribution was not changed by MARF1 knockdown. There are probably two reasons for this phenomenon. One reason is the timing of *in utero* electroporation. At E14.5, only Satb2^+^ upper layer neurons are derived from radial glia and astrocyte production follows a few days later^1^. Therefore, during this transition phase from neurogenesis to gliogenesis, it seems that sMARF1 modulates only Satb2^+^ upper layer neuronal numbers, not deep layer neurons. Another reason may be that sMARF1 specifically controls neurogenesis but not neural migration. Therefore, even though neuronal differentiation by electroporation of the sMARF1 shRNA vector delays, the laminar distribution of electroporated cells may not be affected. Subsequent glial differentiation may increase from the increased neural stem pool by knockdown of sMARF1 as previously described^20^. The change of Satb2^+^ cell number in the superficial layer may contribute to the abnormalities of callosal projection to the contralateral hemisphere^1^, although evidence is lacking. Moreover, it is also likely that sMARF1 is involved in the fate specification of cortical neurogenesis. Further experiments such as *in utero* electroporation at various earlier time points (eg. E10.5, 12.5) and comparison of the rate of neurogenesis of deep layer neurons and superficial neurons would be necessary to clarify the sMARF1 function in brain development.

What molecules could be targeted by sMARF1? Recent advances in sequencing technology, such as the photoactivatable ribonucleoside-enhanced crosslinking and immunoprecipitation (PAR-CLIP) method^21^, enable systematic identification of the targets of RBPs. Conventional RNA immunoprecipitation (RIP) can also identify stably bound RNA targets^22^. Our findings suggest that *sMarf1* expression increases as neuronal differentiation progresses (Fig. 1c). Thus, the target molecule of sMARF1 could be a molecule that progenitor cells but not neurons require for development. These molecules could be stem cell maintenance factors, such as *Sox2* and *Pax6*, or proneural genes that only immature neurons require, such as NeuroD and Ascl family^23^. Further examination is required using the abovementioned methods. MARF1 belongs to the NYN/PIN-like domain, RNase domain family, but the function of the NYN/PIN-like domain is still unclear^17^. Therefore, to elucidate the role of MARF1, we compared the NYN/PIN-like domain of MARF1 with other family members.

Another well-characterized NYN/PIN-like domain-containing protein is Zc3h12a (Regnase-1)^24^. Regnase-1 is a PIN domain-harboring RNase that is critical for preventing severe autoimmune inflammatory disease in mice by destabilizing inflammation-related mRNAs, including *Il6* and *Reg1* itself^24, 25^. The PIN domain shares characteristics with the NYN domain^17^, and Zc3h12a is now categorized as a member of the Zc3h12 gene family, which harbors the NYN domain^26^. In addition, RDE-8 in *Caenorhabditis elegans* encodes a Zc3h12a-like endoribonuclease required for RNA degradation, which functions as a mechanism of RNA interference^27^. Regnase-1 overexpression does not rescue the phenotype associated with the disruption of RDE-8 in *Caenorhabditis elegans* ^27^. These results suggest that NYN/PIN-like domains are diverse. Based on information from the protein database InterPro (http://www.ebi.ac.uk/interpro/), the NYN/PIN-like domain also shows significant similarity with the PIN domain of the N-termini of 5′-3′-exonucleases, such as SMG5/6. In eukaryotes, PIN domains are ribonucleases involved in nonsense-mediated mRNA decay^28^ and in the processing of 18 S ribosomal RNA^29^. Taken together, the results suggest that the N-terminal sequence of MARF1 likely displays similar RNase activity or a function in mRNA decay similar to Zc3h12a, RDE-8, and SMG5/6.

Although our results indicate that sMARF1 regulates differentiation through its RNase activity, it is possible that our deletion mutant was inactivated by protein structure changes. Therefore, other experiments, such as introducing point mutations in critical amino acids within the NYN/PIN-like domain, are necessary to confirm our observations. The NYN/PIN-like domain shares a common set of four amino acids with previously characterized nuclease domains^30^. The common amino acids bind to Mg^2+^ ions and are essential for nuclease activity^30^. Thus, substitutions in these common amino acids may help determine the importance of sMARF1 RNase activity in neuronal differentiation.

Recent studies have demonstrated a relationship between abnormal cortical neurogenesis and neurodevelopmental disorders. Rodents lacking the neurogenesis-related genes *Pax6* or *Tbr2* exhibit impaired neurogenesis and behavioral abnormalities associated with an autistic phenotype^31^ and hyperactivity^32^. Overproduction of upper-layer neurons induced by drug injection leads to autism-like behaviors in mice^33^. These findings support the hypothesis that normal cortical neurogenesis is critical for the development of the intact brain. In addition, copy number variation (CNV) is a potential risk factor for neurodevelopmental disorders^34^. Chromosome 16p13.11 is known to have CNVs associated with neurodevelopmental disorders^34–37^. We found that miR-484, which is encoded in the core locus of the chromosome 16p13.11 CNV, regulates cortical neurogenesis^18^. *Marf1* is also located on chromosome 16p13.11 contiguous to miR-484^18^. Taken together, these findings suggest that sMARF1 may have synergistic effects with miR-484 on neurogenesis as candidate genes of 16p13.11 CNV. Therefore, future studies of sMARF1 will hopefully uncover not only the detailed mechanisms of sMARF1 functions but also the relationships between sMARF1 and neurodevelopmental disorders.

## Materials and Methods

This study was approved by the institutional committee of Osaka University.

### Animals

Slc-ICR mice were used in this study, and all mice were purchased from SLC Japan. Mice were euthanized with an overdose of a mixture of 0.5 mg/ml Vetorphale (Meiji Seika Pharma), 0.4 mg/ml Dormicum (Roche), and 0.03 mg/ml Domitor (Orion Pharma) by peritoneal injection. All procedures complied with the Osaka University Medical School Guidelines for the Care and Use of Laboratory Animals.

### RT-PCR

Cells and tissues were homogenized in TRIzol reagent (Thermo Fisher Scientific). Isolated total RNA was purified with an RNeasy Micro kit (QIAGEN). The complementary DNA (cDNA) was synthesized with a High Capacity cDNA Reverse Transcription kit (Applied Biosystems). A no template reaction was used as a negative control. Mouse ovary tissue containing cumulus cells and oocytes was used as a positive control. Primers for Marf1 exon3 were as follows: Forward: 5′-TTCACCAAGATAATGATGCTAAGC-3′, Reverse: 5′-TTTTCCATGCCTTTTGTTCC-3′.

### Real-time PCR analysis

TaqMan real-time PCR analysis was conducted using TaqMan Universal Master Mix II (Thermo Fisher Scientific) with specific probe mixtures for each gene (*Marf1*: Mm00463593_m1, glyceraldehyde-3-phosphate dehydrogenase (*Gapdh*): Mm99999915_g1). The expression of each gene was normalized to *Gapdh*. The reaction and the subsequent quantification were conducted using QuantStudio 7 Flex Real-Time PCR System (Applied Biosystems).

### Plasmids

The sMarf1 open reading frame (ORF) was amplified from cDNA obtained from mouse cortical neurons using specific primers (Forward: 5′-CCATATTGTCTGGCTATGT-3′, Reverse: 5′-TCCATGTTCAAATGGGAG-3′). Then, cDNA was cloned into a pCR Blunt II TOPO vector (Thermo Fisher Scientific) with the restriction enzyme sites NotI and XhoI. Next, the sMarf1 ORF was inserted into a pCAGIG (Addgene) vector by restriction enzyme digestion using a DNA ligation kit ver. 2.1 (TaKaRa). To add the V5-tag to the C-terminus of sMarf1, the sMarf1 ORF was subcloned into a pcDNA3.1/V5-His TOPO vector (Thermo Fisher Scientific). In parallel, to establish the ΔNYN sMarf1 construct, the sMarf1 ORF was amplified using specific primers (Forward: 5′-CACCATGGGCCACACTCTACTCTAT-3′, Reverse: 5′-TTTAAGCTTGGTTACAGGTGCAAAAG-3′), and the product was cloned into the pcDNA 3.1/V5-His TOPO vector. Finally, to express the proteins in neuronal cells, both reading frames were transferred to a pCAGIG vector containing a V5 epitope tag. For the construction of the sMarf1 shRNA vector, oligos (Sense: 5′-GATCCCCACACCTCACTTGTGCACCATTCAAGAGATGGTGCACAAGTGAGGTGTTT-3′, Anti-sense: 5′-AGCTTAAAAAACACCTCACTTGTGCACCATCTCTTGAATGGTGCACAAGTGAGGTGT-3′) were designed using OligoEngine software. The oligos were annealed and cloned into a pSUPER retro.neo + gfp vector (Addgene).

### Immunocytochemistry

Cells were fixed for 10 min in 4% paraformaldehyde in 0.1 M phosphate buffer (PB). Permeabilization was performed for 10 min in 0.2% NP-40. The cells were blocked for 1 h in 0.5% bovine serum albumin (BSA) and 6% normal goat serum in phosphate-buffered saline (PBS) (-). The primary antibodies (mouse and rabbit anti-Tuj1 (1:1000, Covance), mouse anti-Ki67 (1:200, BD Bioscience), and rabbit and chicken anti-green fluorescent protein (GFP) (1:400, Thermo Fisher Scientific)) were incubated with the samples overnight at 4 °C. After washing, the secondary antibodies (Alexa 488 anti-chicken and anti-rabbit immunoglobulin G (IgG) (1:500, Thermo Fisher Scientific), and Alexa 568 anti-rabbit and anti-mouse IgG (1:500, Thermo Fisher Scientific) were incubated with the samples for 1 h at room temperature. After washing away unbound antibody, the cells were counterstained with 4′,6-diamidino-2-phenylindole (DAPI) (1:1000, Dojindo). Finally, the slides were mounted with Fluorescent Mounting Medium (Dako). Fluorescence images were acquired using a BX51 fluorescence microscope (Olympus).

### Immunohistochemistry

Post-fixed E14.5 or electroporated mouse brains were cut into 20-μm sections. Sections were fixed for 15 min in 4% paraformaldehyde in 0.1 M PB followed by blocking and permeabilization with 10% BSA and 0.3% Triton X-100. Then, the tissue was treated with a M.O.M. blocking kit according to the manufacturer’s instructions (Vector Laboratories). Rabbit anti-MARF1 (1:100, a gift from Dr. Su^16^), mouse anti-MAP2 (1:1000, Sigma), rabbit anti-Tbr2 (1:500, Abcam), mouse anti-Satb2 (1:200, Abcam), mouse anti-Ki67 (1:200, BD Biosciences), rabbit anti-Pax6 (1:500, Millipore), rabbit anti-GFP (1:800, Thermo Fisher Scientific), and chicken anti-GFP (1:500, Thermo Fisher Scientific) were used for primary antibodies. Cy3-conjugated streptavidin (1:1000, Jackson ImmunoResearch Laboratories), Alexa 488-conjugated anti-rabbit IgG (1:500, Thermo Fisher Scientific), Alexa 568-conjugated anti-rabbit IgG (1:500, Thermo Fisher Scientific), and Alexa 488-conjugated anti-chicken IgY (1:500, Abcam) were used as secondary antibodies. DAPI (1:1000, Dojindo) was used for nuclei counterstaining. For quantitative analysis, sections with similar anatomical GFP distributions were selected for analysis, and then, a total of 3–5 sections were analyzed per embryo using an FV-1200 laser-scanning confocal microscope (Olympus). The subventricular zone area was determined by DAPI staining^38^.

### Immunohistochemistry signal amplification and same animal species antigen

The TSA Plus Fluorescein System (PerkinElmer) was used to amplify the immunohistochemistry signal. After fixing for 15 min in 4% paraformaldehyde in 0.1 M PB, sections were incubated in 0.3% H_2_O_2_ in methanol to block endogenous peroxidase activity for 30 min at room temperature. Then, the sections were incubated in sodium citrate buffer (10 mM sodium citrate, 0.05% Tween-20, pH 6.0) for 30 min at 70 °C in an autoclave for antigen retrieval. Thereafter, the samples were immediately washed with PBS(-) and incubated for 1 h in blocking buffer containing 10% goat serum, 5% BSA and 0.3% Triton X-100 at RT. Rabbit anti-MARF1 (1:100, from Dr. Eppig^16^) was used as the primary antibody. The secondary antibody reaction was performed for 30 min at room temperature with horseradish peroxidase-conjugated antibody (1:2000, Cell Signaling Technology) in blocking buffer. The sections were washed in TNT buffer (0.1 M Tris-HCl pH 7.5, 0.15 M NaCl, 0.05% Tween-20) three times for 5 min each. After the TNT buffer was removed, the sections were incubated in TSA Amplification Reagents for 7 to 10 min. Then, the sections were washed in TNT buffer three times for 5 min each. Finally, the sections were incubated in the sodium citrate buffer for 15 min at 95 °C in the autoclave to inactivate the rabbit anti-MARF1 antibody. Washed samples were incubated in the blocking buffer for 1 h at room temperature. The samples were used for standard immunohistochemistry.

### Western blotting

All cells and tissues were lysed in radioimmunoprecipitation assay buffer (50 mM Tris-HCl pH 8, 150 mM NaCl, 1% NP-40, 0.1% sodium dodecyl sulfate (SDS), 0.5% sodium deoxycholate) containing protease inhibitors (Roche). Lysates were loaded onto 8% SDS-polyacrylamide gels and were transferred to polyvinylidene fluoride membranes. Membranes were blocked for 1 h in 5% skim milk in PBS-T (0.05% Tween-20). The primary antibodies (rabbit anti-MARF1 (1:3000, from Dr. Eppig^16^), mouse anti-V5 (1:2000, Thermo Fisher Scientific), and rabbit anti-β-Actin (1:3000, Cell Signaling Technology)) were diluted in blocking buffer to the indicated concentrations and were incubated with membranes overnight at 4 °C. The secondary antibody reaction was performed for 1 h at room temperature with horseradish peroxidase-conjugated antibody (1:2000, Cell Signaling Technology) in blocking buffer. Enhanced chemiluminescence (GE Healthcare) was used for signal detection. All images were obtained using an LAS-3000 image analyzer (Fujifilm). Lysate obtained from HEK293 cells transfected with an sMARF1 expression vector was used as the positive control for the anti-MARF1 antibody. Empty pCAGIG vector-transfected E15.5 neuron lysates were used as a negative control for the anti-V5 antibody.

### Cortical progenitor cell culture and transfection

Cortical progenitor cells were isolated from E12.5 mice. Isolated cortices were transferred to Neurobasal medium (Thermo Fisher Scientific) containing 40 ng/ml fibroblast growth factor 2 (Promega), 2% B27 (Thermo Fisher Scientific), 120 mg/ml penicillin, 200 mg/ml streptomycin sulfate, and 600 mg/ml glutamine. The cells were plated on poly-D-lysine-coated (Sigma) and laminin-coated (BD) 4-well chamber slides at a density of 4.0 × 10^5^ cells/well and were maintained at 37 °C in the presence of 5% CO_2_. For transfections, medium was mixed with 2 μg of plasmid DNA or shRNA vector and 1.5 μl of FuGENE HD (Promega) in 100 μl per well for 15 min at room temperature, after which the transfection mixture was applied to the chamber slides.

### Cortical neuron culture and nucleofection

E15.5 mouse cortices were digested in 0.25% trypsin (Thermo Fisher Scientific) supplemented with DNase (1:1000, Sigma) for 20 min at 37 °C. Cells were dissociated in DMEM containing 10% fetal bovine serum (FBS, Thermo Fisher Scientific), 120 mg/ml penicillin, and 200 mg/ml streptomycin sulfate. Then, cells were washed and resuspended in 100 μl of Mouse Neuron Nucleofector Solution (Lonza) containing 3 μg of plasmid DNA or shRNA vector. After electroporation using a Nucleofector kit (Lonza), the cells were immediately mixed with 500 μl of DMEM/F-12 containing 10% FBS, and the cells were plated onto poly-L-lysine-coated dishes and cultured at 37 °C in the presence of 5% CO_2_. The medium was exchanged with DMEM/F-12 (Thermo Fisher Scientific) containing 2% B27 supplement (Thermo Fisher Scientific) 12 h later.

### *In utero* electroporation

*In utero* electroporation was performed using a square-wave electroporator (CUY21SC, NEPAGENE) that delivered five 50-ms pulses of 30 V with 950-ms intervals, as previously described^18, 19^. E13.5 or E14.5 mice were injected with 6.0 μg of plasmid DNA in 2 μl of diluted water. For sMARF1 shRNA vector injection, we mixed nuclear EGFP with the solution at a 1:3 ratio. Trypan blue (1%, Gibco) was co-injected as a tracer.

### Cell counting and quantification

For the *in vitro* experiments, 100 GFP^+^ transfected cells/well in 4-well chamber slide were counted as n = 1. Three to four (n = 3–4) independent experiments were performed. In cell death assay, we counted the cells having fragmented or condensed nucleus as apoptotic cells, as described^18^. For the *in vivo* experiments, three sets of 100 GFP^+^ electroporated cells/brain section were counted as n = 1. n = 3 for individual treated group. Three control embryos and three conditional embryos were selected using similar electroporation conditions and were analyzed for each of the experiments.

### Statistical analysis

Results were analyzed using Student’s *t* tests or one-way ANOVA followed by Tukey-Kramer tests. All data are presented as the mean ± SEM.

## Electronic supplementary material

Supplementary Figures
