# Supplementary material for: The RNA-binding protein MARF1 promotes cortical neurogenesis through its RNase activity domain
Source: Sci Rep. 2017 Apr 25;7:1155. doi: 10.1038/s41598-017-01317-y (PMC5430739; doi:10.1038/s41598-017-01317-y)
Supplement: Supplementary file 1 — Supplementary Figures [file 41598_2017_1317_MOESM1_ESM.pdf]

Supplementary Information

The RNA-binding protein MARF1 promotes cortical neurogenesis through its RNase activity domain

Yoshitaka Kanemitsu[1,2], Masashi Fujitani[3,4,5 *], Yuki Fujita[3], Suxiang Zhang[3], You-Qiang Su[6], Yukio Kawahara[7], Toshihide Yamashita[1,3*]

1, Department of Molecular Neuroscience, Graduate School of Frontier Biosciences, Osaka University, 1-3 Yamadaoka, Suita, Osaka 565-0871, Japan

2, Interdisciplinary Program for Biomedical Sciences, Osaka University, 2-2 Yamadaoka, Suita, Osaka 565-0871, Japan

3, Department of Molecular Neuroscience, Graduate School of Medicine, Osaka University, 2-2 Yamadaoka, Suita, Osaka 565-0871, Japan

4, Molecular Research Center for Children' s Mental Development, United Graduate School of Child Development,Osaka University, 2-2 Yamadaoka, Suita, Osaka 565-0872, Japan

5, Department of Anatomy and Neuroscience, Hyogo College of Medicine 1-1, Mukogawa-cho, Nishinomiya, Hyogo 663-8501, Japan

6, State Key Laboratory of Reproductive Medicine, Nanjing Medical University, 140 Hanzhong Road, Nanjing 210029, Jiangsu Province, China

7, Department of RNA Biology and Neuroscience, Graduate School of Medicine, Osaka University, 2-2 Yamadaoka, Suita, Osaka 565-0871, Japan

Supplementary Figure 1

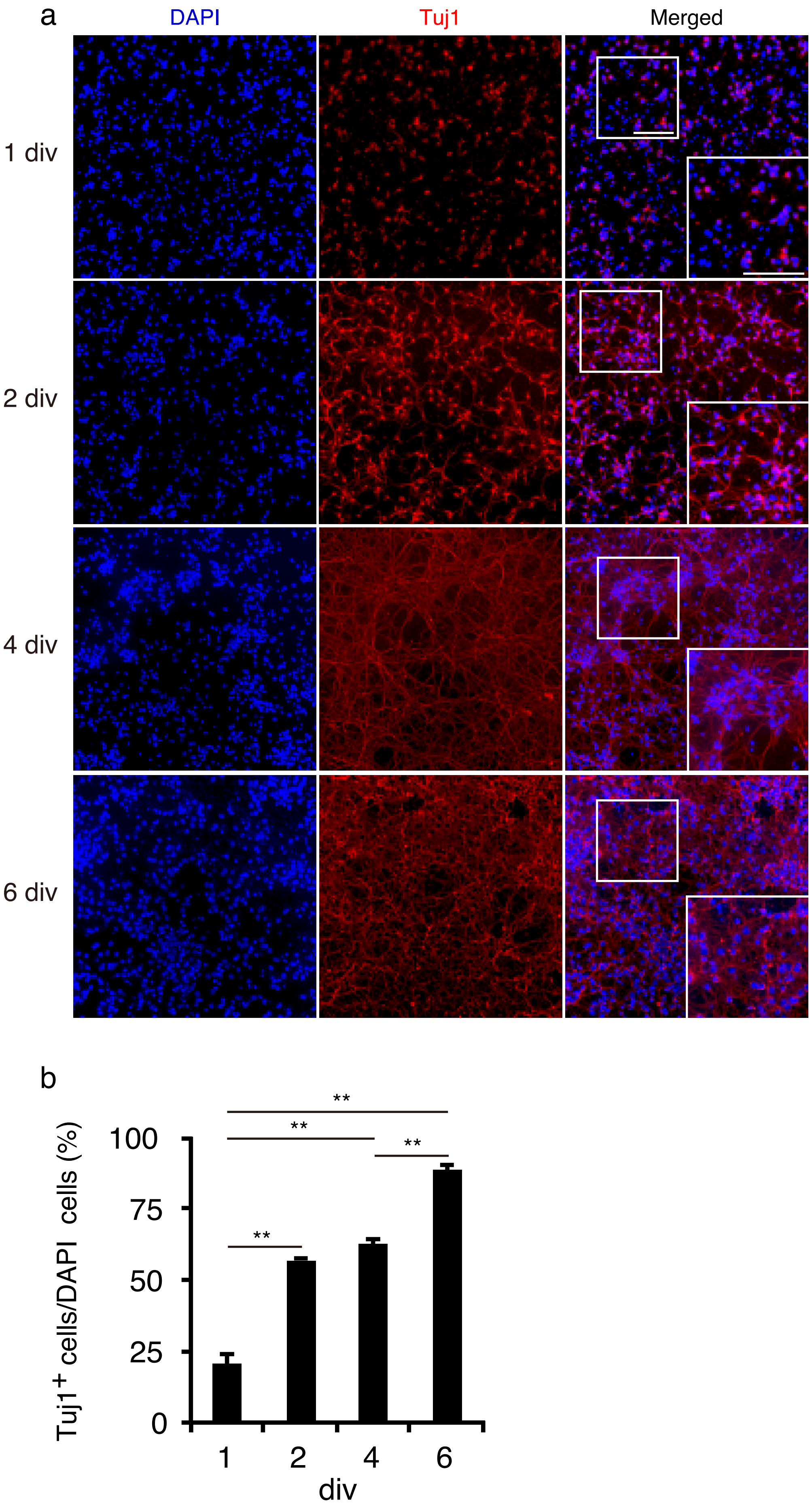

Supplementary Figure 1: Confirmation of full maturation of E12.5 cortical progenitor cells after 6 days culture, and confirmation of knockdown of MARF1 *in vivo* by shRNA vector.
(a) Immunostaining for DAPI (blue) and Tuj1 (red) in E12.5 cortical progenitor cells cultured for 1, 2, 4, 6 days *in vitro* (div). Scale bar, 100 μm. (b) Quantification of percentage of Tuj1$^+$ living neurons. *p<0.05, **p<0.01 (n=3, one-way ANOVA Tukey-Kramer test).

Supplementary Figure 2

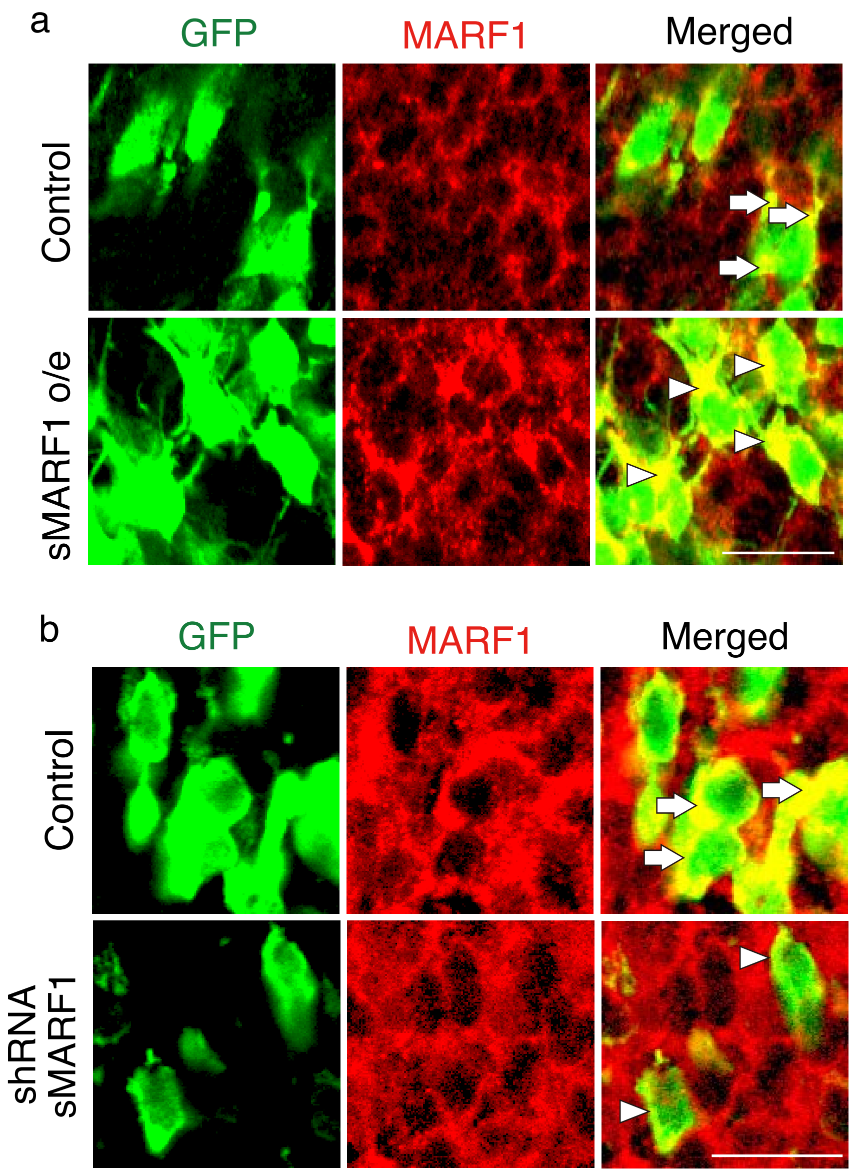

Supplementary Figure 2: Confirmation of overexpression and knockdown of MARF1 *in vivo*.
(a) Immunostaining for GFP (green) and MARF1 (red) in the E16.5 mouse cortex 3 days after electroporation with Control or Marf1 expression vector (sMARF1 o/e). White arrows indicate GFP^+ and $MARF1^+$ cells. White arrowheads indicate MARF1 overexpressed GFP^+ cells. Scale bar, 20 μm.
(b) Immunostaining for GFP (green) and MARF1 (red) in the E16.5 mouse cortex 3 days after electroporation with scrambled (Control) or Marf1-specific shRNA vector (sMARF1 shRNA). White arrows indicate GFP^+ and $MARF1^+$ cells. White arrowheads indicate MARF1 knockdown GFP^+ cells. Scale bar, 20 μm.

Supplementary Figure 3

E12.5

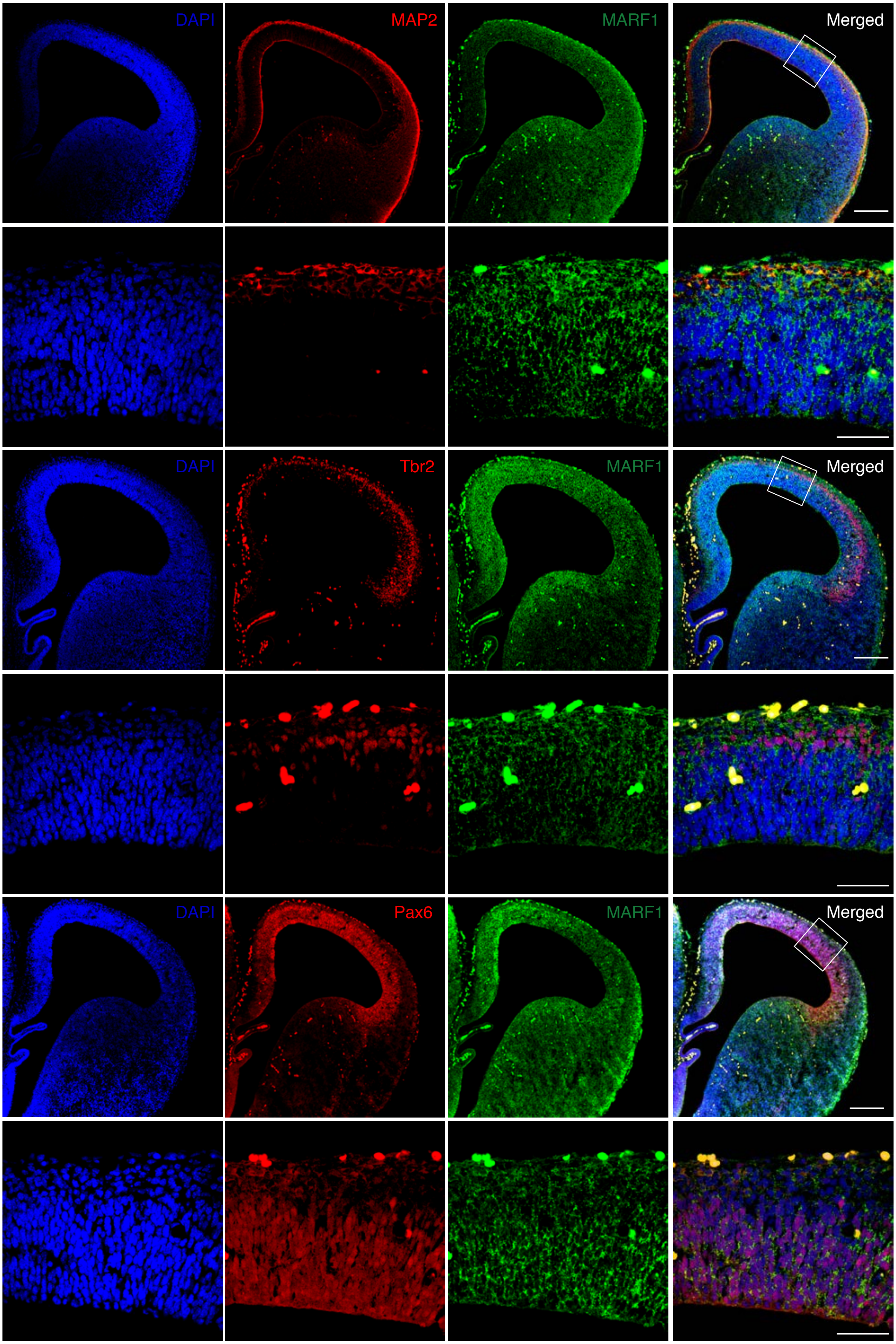

Supplementary Figure 3: sMARF1 expression in E12.5 mouse cortex.
Immunostaining for MARF1 (green), DAPI (blue), and neuronal marker, MAP2 (red), basal progenitor marker, Tbr2 (red) or radial glial marker, Pax6 (red) in coronal sections of the E12.5 mouse cortex.
Scale bar, 200 μm in the merged images, 50 μm in the magnified images.

Supplementary Figure 4

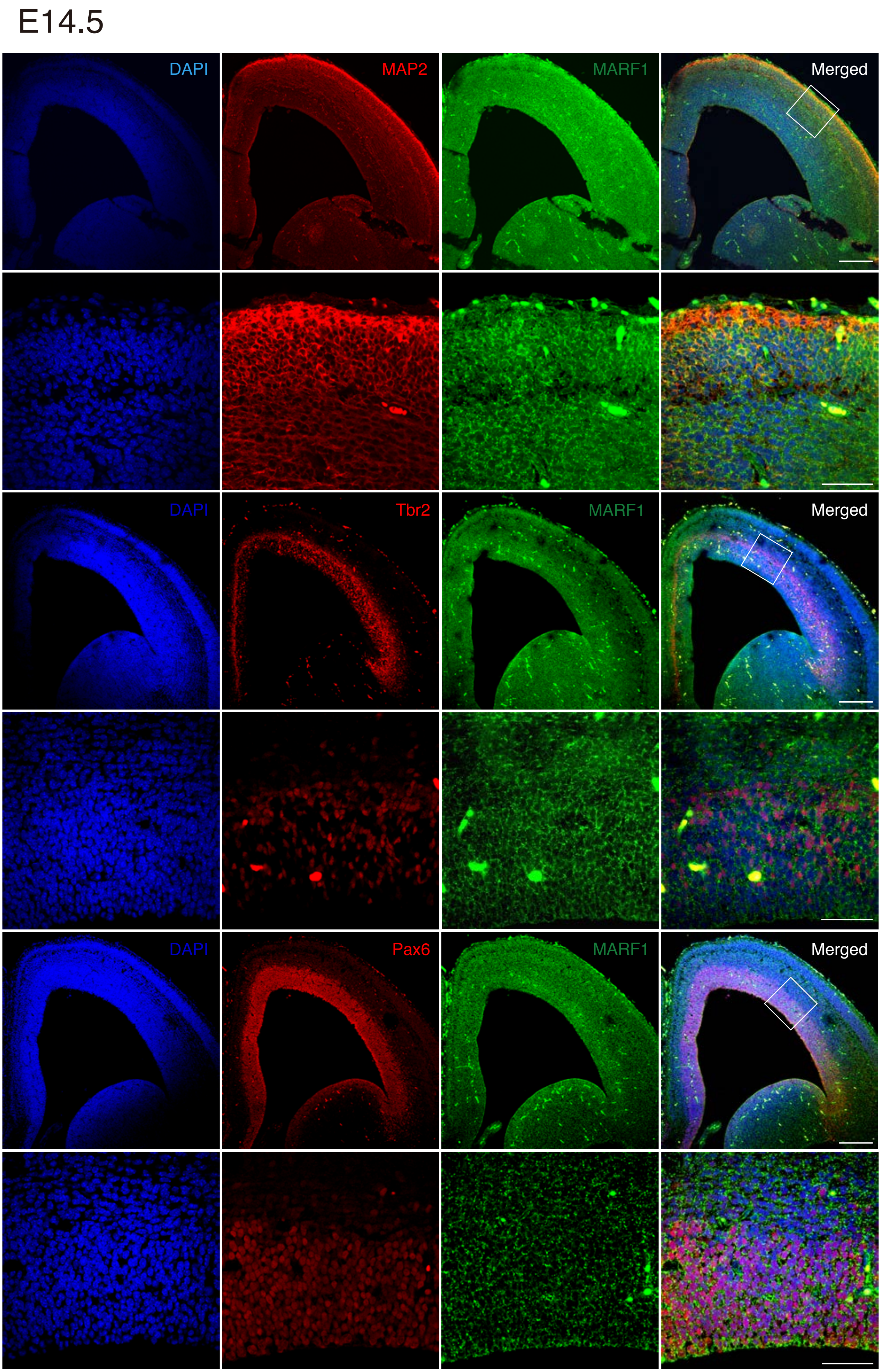

Supplementary Figure 4: sMARF1 expression in E14.5 mouse cortex.
Immunostaining for MARF1 (green), DAPI (blue), and neuronal marker, MAP2 (red), basal progenitor marker, Tbr2 (red) or radial glial marker, Pax6 (red) in coronal sections of the E14.5 mouse cortex.
Scale bar, 200 μm in the merged images, 50 μm in the magnified images.

Supplementary Figure 5

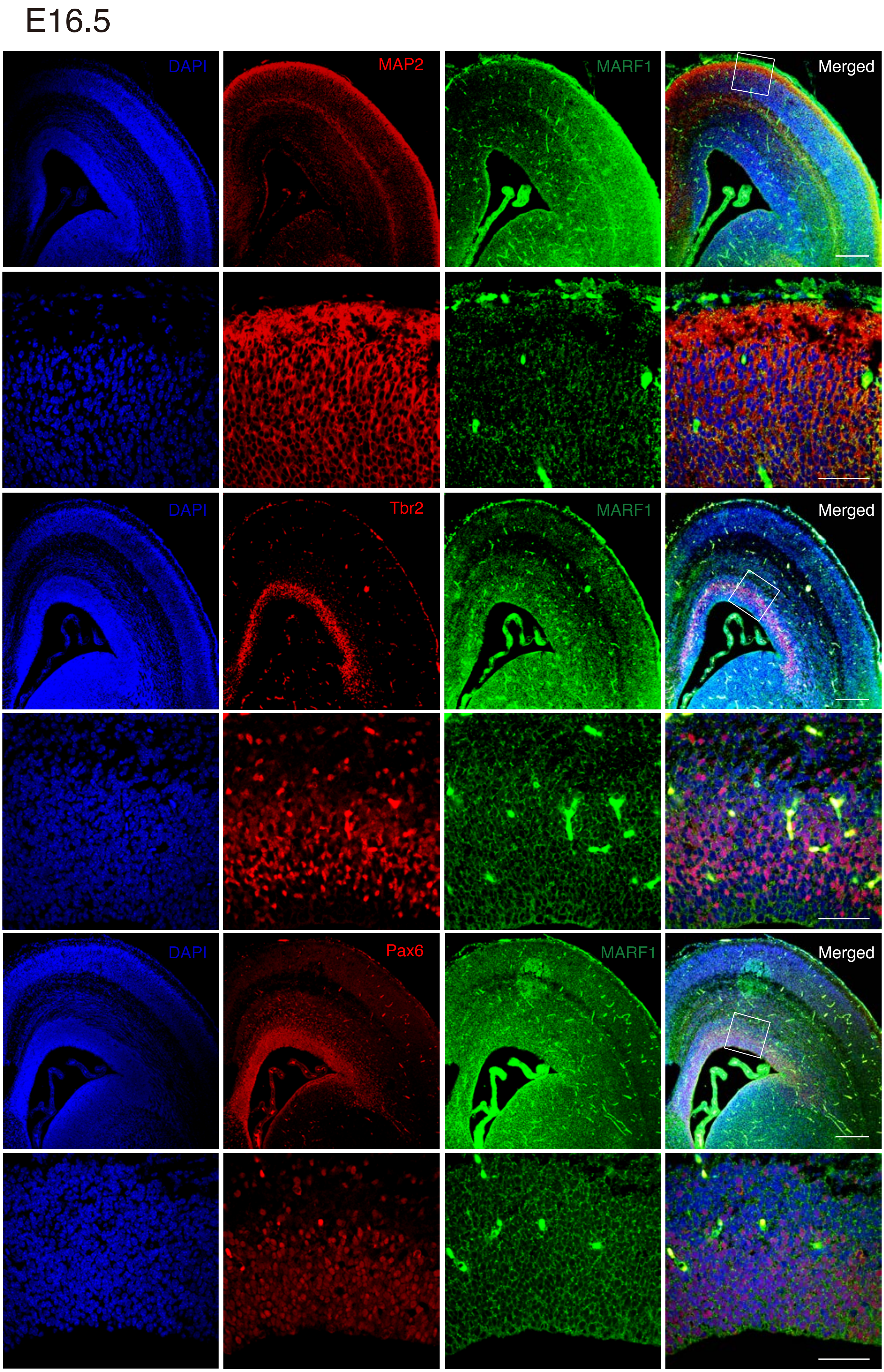

Supplementary Figure 5: sMARF1 expression in E16.5 mouse cortex.
Immunostaining for MARF1 (green), DAPI (blue), and neuronal marker, MAP2 (red), basal progenitor marker, Tbr2 (red) or radial glial marker, Pax6 (red) in coronal sections of the E16.5 mouse cortex.
Scale bar, 200 μm in the merged images, 50 μm in the magnified images.

Supplementary Figure 6

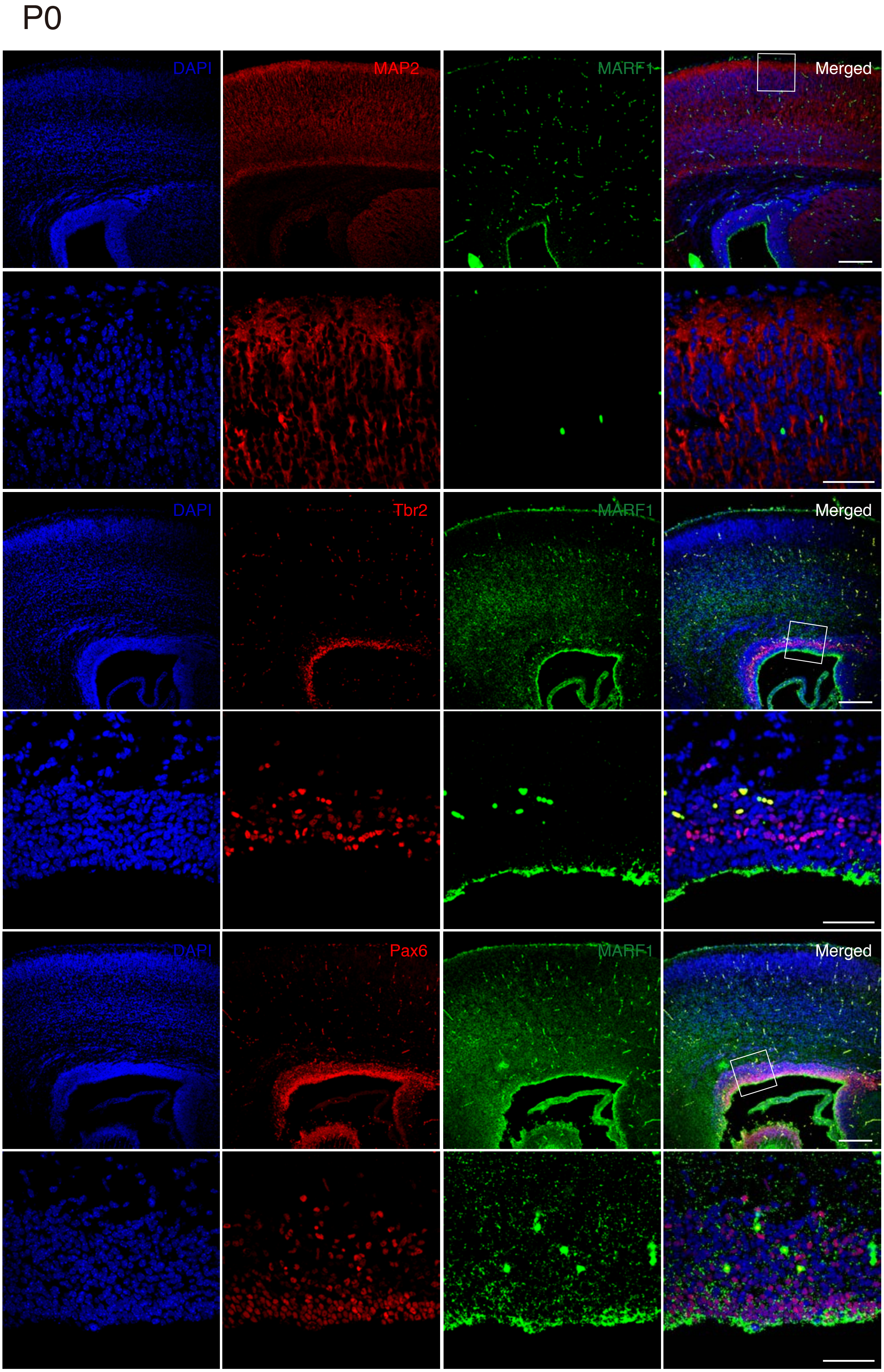

Supplementary Figure 6: sMARF1 expression in P0 mouse cortex.
Immunostaining for MARF1 (green), DAPI (blue), and neuronal marker, MAP2 (red), basal progenitor marker, Tbr2 (red) or radial glial marker, Pax6 (red) in coronal sections of the P0 mouse cortex.
Scale bar, 200 μm in the merged images, 50 μm in the magnified images.

Supplementary Figure 7

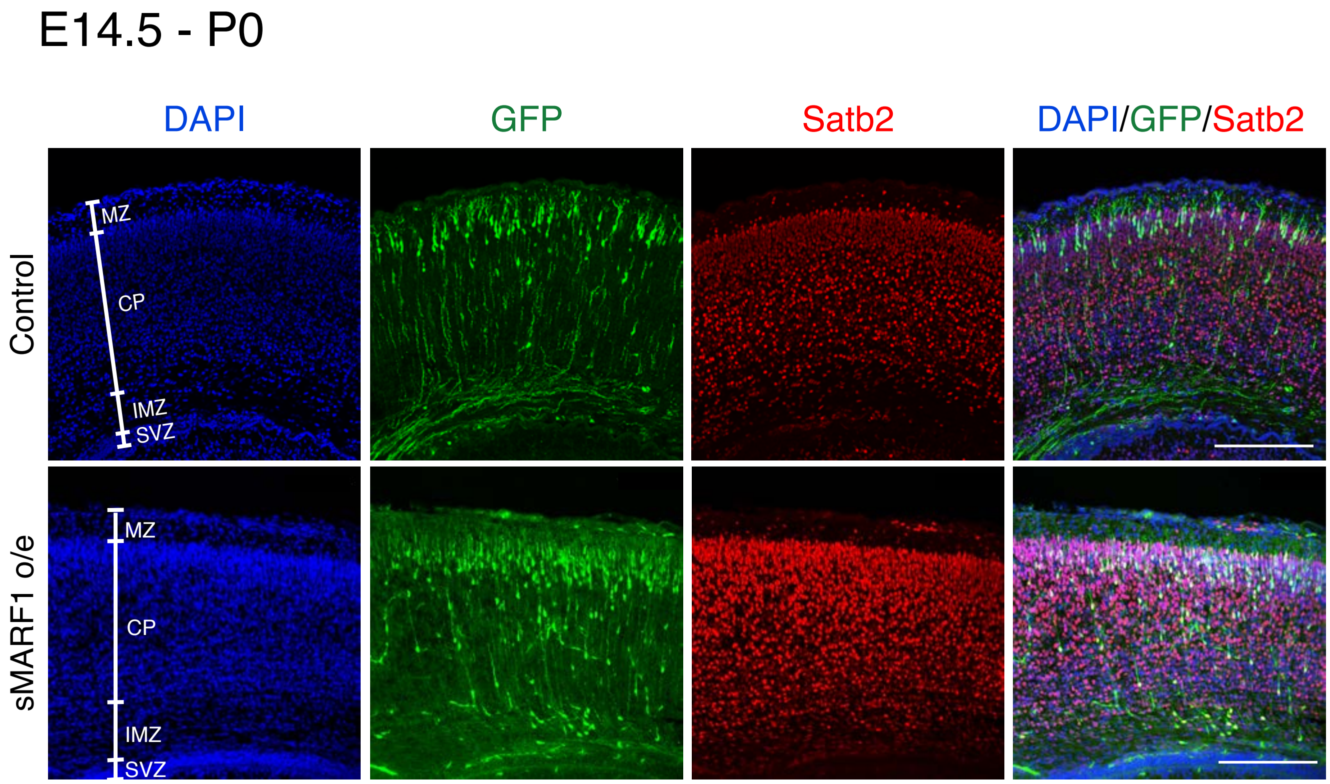

Supplementary Figure 7: sMARF1 overexpression have no effect on laminar distribution of electroporated neurons.

(a) Immunostaining for DAPI (blue), GFP (green) and Satb2 (red) in P0 mouse cortices 5 days after electroporation with pCAGIG (Control) or sMarf1 expression vector (sMARF1 o/e). Scale bar, 200μm. MZ: marginal zone, CP: cortical plate, IMZ: intermediate zone, SVZ: subventricular zone

Supplementary Figure 8

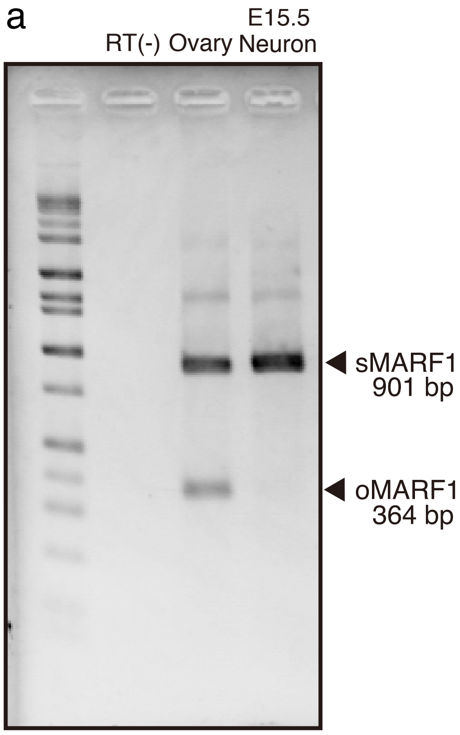

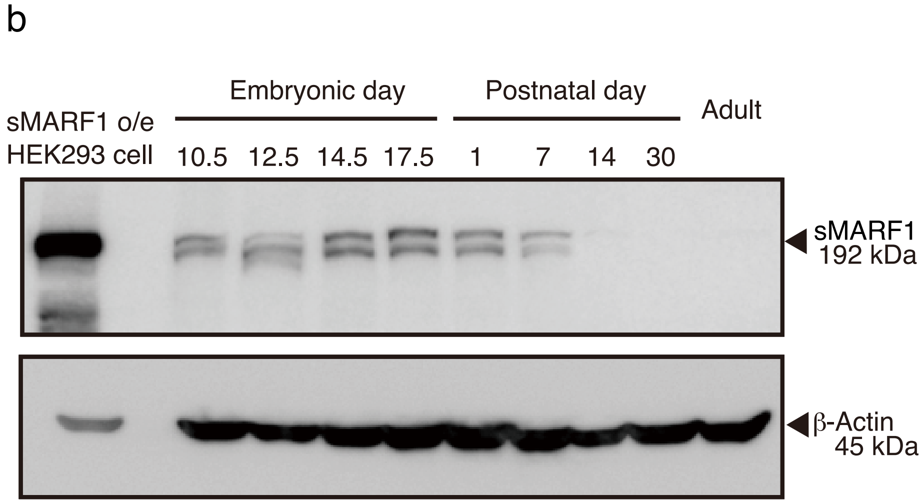

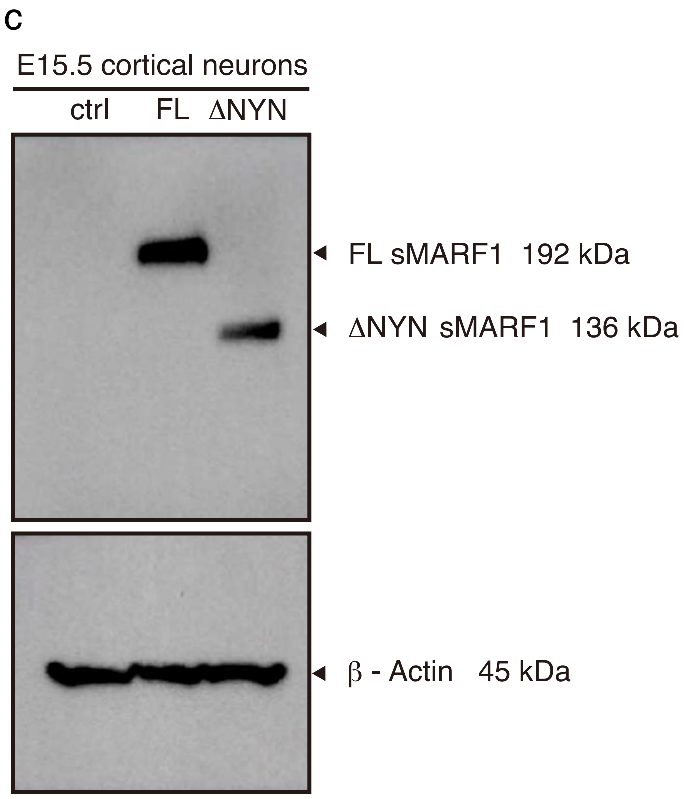

Supplementary Figure 8:
The full-length images of gel and blotting.

(a) RT-PCR analysis for exon3 of Marf1mRNA in mouse ovary tissue and E15.5 cultured neurons. The full-length image of Figure 1a.
(b) Western blotting for sMARF1 in mouse cortical lysated at the embryonic, postnatal, and adult ages. The same image as Figure 1b, because membrane was cut before incubating with antibodies.
(c) Western blotting for V5 epitope in E15.5 cortical neurons transfected with pCAGIG (ctrl), FL sMARF1 expression vector (FL), or ΔNYN sMARF1 expression vector (ΔNYN). The full-length image of Figure 4c.
